# Resistance and resilience of root fungal communities to water limitation in a temperate agroecosystem

**DOI:** 10.1002/ece3.2900

**Published:** 2017-04-05

**Authors:** Jessie R. Furze, Adam R. Martin, Joshua Nasielski, Naresh V. Thevathasan, Andrew M. Gordon, Marney E. Isaac

**Affiliations:** ^1^ Department of Physical and Environmental SciencesUniversity of Toronto ScarboroughTorontoOntarioCanada; ^2^ Department of Physical and Environmental Sciences and the Centre for Critical Development StudiesUniversity of Toronto ScarboroughTorontoOntarioCanada; ^3^ Department of GeographyUniversity of TorontoTorontoOntarioCanada; ^4^ School of Environmental SciencesUniversity of GuelphGuelphOntarioCanada

**Keywords:** agroforestry, arbuscular mycorrhizal fungi, *Glycine max*, rainfall reduction, tree‐based intercropping, T‐RFLP, water limitation

## Abstract

Understanding crop resilience to environmental stress is critical in predicting the consequences of global climate change for agricultural systems worldwide, but to date studies addressing crop resiliency have focused primarily on plant physiological and molecular responses. Arbuscular mycorrhizal fungi (AMF) form mutualisms with many crop species, and these relationships are key in mitigating the effects of abiotic stress in many agricultural systems. However, to date there is little research examining whether (1) fungal community structure in agroecosystems is resistant to changing environmental conditions, specifically water limitation and (2) resilience of fungal community structure is moderated by agricultural management systems, namely the integration of trees into cropping systems. Here, we address these uncertainties through a rainfall reduction field experiment that manipulated short‐term water availability in a soybean‐based (*Glycine max* L. Merr.) agroforest in Southern Ontario, Canada. We employed terminal restriction fragment length polymorphism analysis to determine the molecular diversity of both general fungal and AMF communities in soybean roots under no stress, stress (rainfall shelters added), and poststress (rainfall shelters removed). We found that general fungal and AMF communities sampled from soybean roots were resistant to rainfall reduction in a monoculture, but not in an agroforest. While AMF communities were unchanged after stress removal, general fungal communities were significantly different poststress in the agroforest, indicating a capacity for resiliency. Our study indicates that generalist fungi and AMF are responsive to changes in environmental conditions and that agroecosystem management plays a key role in the resistance and resilience of fungal communities to water limitation.

## 1 Introduction

There is considerable uncertainty regarding how climate change and associated expected shifts in temperature and precipitation regimes will influence agricultural crop production worldwide (Lobell & Field, 2007; Schmidhuber & Tubiello, 2007; Ziervogel & Ericksen, 2010). On the one hand, certain models predicting crop growth and yield from physiological traits suggest that increased growing season temperatures, coupled with more variable and less evenly distributed precipitation, may result in net decreases in crop yields of 1%–13% (Nelson et al., 2010). Alternatively, other models predict increased crop yields in certain regions in response to warmer conditions and extended growing seasons (Smith et al., 2013), coupled with elevated CO_2_ concentrations that may increase crop water use efficiency and biomass accumulation; however, these responses may differ widely (at least) between C3 and C4 crops (McGrath & Lobell, 2013; Yang et al., 2014). Although a consensus understanding of how climate change will influence crop growth and yield remains elusive, there is considerable interest and urgency for understanding the mechanisms by which crops will respond to changing climate, in order to develop agricultural adaptation strategies (Beebe et al., 2011).

In seeking to understand the impacts of climate change on crops, researchers have largely focused on elucidating the physiological characteristics that mechanistically underpin plant resiliency to environmental stress (Araújo et al., 2015). In agricultural systems, there have been considerable efforts in understanding how crop physiology changes in response to shifts in water availability (e.g., Nasielski et al., 2015), temperature (e.g., Prasad, Boote, Allen, & Thomas, 2002), salinity (e.g., Conde, Chaves, & Geros, 2011), atmospheric CO_2_ concentrations (e.g., Prasad et al., 2002), and changes in soil chemistry (e.g., Lynch, 2011). For example, as the world's leading economic oilseed crop and vegetable protein, soybean (*Glycine max* L. Merr.) has been one of the most well‐studied crops with respect to environmental change (Manavalan, Guttikonda, Tran, & Nguyen, 2009). Studies on soybean have examined how nearly all aspects of soybean physiology and reproductive biology respond to abiotic stress, including phenology (e.g., Liu, Anderson, & Jensen, 2003), pod abortion and expansion (e.g., Liu et al., 2003; Liu, Jensen, & Andersen, 2004), yield (e.g., Desclaux, Huynh, & Roumet, 2000), seed mass (e.g., Desclaux & Roumet, 1996; Araújo et al., 2015), and yield stability (e.g., Nasielski et al., 2015).

Although such crop‐specific physiological studies have been crucial in understanding and predicting agricultural resiliency to environmental change, there remains a surprising lack of information on how shifts in climate will affect plant‐microbial mutualisms: a key dimension of agroecological dynamics that has critical implications for crop growth and yield under changing environmental conditions (Compant, van der Heijden, & Sessitsch, 2010). It is widely hypothesized that plant‐microbial mutualisms, particularly those between crops and arbuscular mycorrhizal fungi (AMF), enhance crop resistance and resilience to biotic and abiotic stresses for a range of plant species (Koltai & Kapulnik, 2010). (Although definitions differ, here we refer to resistance as the capacity of a system to remain in a stable state in response to a disturbance, while resilience refers to the capacity of a system to return to that stable state after a temporary shift away from that state in response to a disturbance (Holling, 1973; Gunderson, Holling, Pritchard, & Peterson, 2002; Griffiths & Philippot, 2012).) For instance, in managed agroecosystems, AMF are critically important in conferring enhanced crop fitness by improving nutrient (especially inorganic phosphorus [P]) uptake (Ryan & Graham, 2002; Smith & Smith, 2011). But despite the well‐documented importance of these mutualisms, there remains little understanding of how fungal communities, and in turn crop‐fungal relationships, may change in response to shifting climate.

Similarly, there are few studies that evaluate how alternative agricultural management systems might result in greater resistance or resilience of AMF communities to environmental change. Agroforestry systems in particular are increasingly viewed as an ecologically robust alternative to conventional monoculture management (Nair, 2007). In temperate systems, studies have shown that the intercropping of annual crops with woody perennials positively influences crop growth and yield by mitigating multiple environmental stresses (Thevathasan & Gordon, 2004; Rivest, Cogliastro, & Olivier, 2009). A few studies have also shown that agroforestry management results in more diverse soil microbial communities (Chifflot, Rivest, Olivier, Cogliastro, & Khasa, 2009; Bainard, Koch, Gordon, & Klironomos, 2012), which ultimately lead to enhanced rates of soil nutrient cycling and soil organic matter decomposition (Bent, 2006; Lugtenberg & Kamilova, 2009; Finzi et al., 2015). However, to our knowledge there are no studies examining whether agroforestry management systems in fact buffer the effects of changing climate on AMF communities, which are likely to be sensitive to changes in temperature or precipitation (Querejeta, Egerton‐Warburton, & Allen, 2009; Compant et al., 2010).

**Figure 1:**
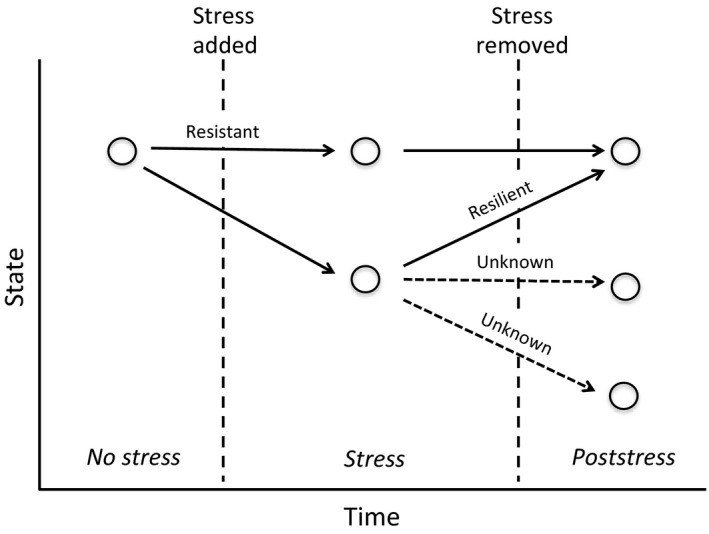
Hypothesized relationships between time and the state of generalist fungi and AMF populations. The *No stress* (first point) is affected by the addition of *Stress* (rainfall reduction) resulting in new states (center points), which are affected by the removal of stress (rainfall reduction removed) resulting in *Poststress* states (last points). Two key dynamics are identified: resistant (no change after stress is added) and resilient (change after stress is applied and recovery after stress is removed). Other generalist fungi and AMF population outcomes are unknown

Using an in situ rainfall reduction experiment in an experimental temperate agroforestry system, coupled with molecular techniques (terminal restriction fragment length polymorphism [T‐RFLP]), we evaluated how water limitation and management influences fungal community structure in agroecosystems. Our analyses were designed to address the following questions: (1) does general fungal and AMF community structure change in response to water limitation? If so, then (2) does agroforestry management enhance the resiliency of general fungal and AMF community structure after water limitation, as compared to conventional monoculture management? We conceptualize several possible changes in the state of fungal communities before and after a stress is added and removed (Figure 1): stress inducing no change in community structure (resistance) and stress removal inducing change in community structure (resilience). In this study, we assume a significant change in community structure after the addition of stress, followed by a significant change in community structure after stress has been removed, indicates a capacity for resiliency. However, we acknowledge that our data highlights community structure and not species identity; therefore, we also depict the possible unknown changes in the state of fungal communities. These unknown changes in the state of fungal communities may alternatively indicate a continuation of changing communities poststress. We hypothesize that (1) due to the sensitivities of AMF to water availability (Querejeta et al., 2009; Compant et al., 2010), AMF communities will not be as resistant to water limitations compared to generalist fungi; and that (2) due to microclimate buffering capacity in agroforestry systems (Jose, 2009; van Noordwijk et al., 2014), general fungal and AMF community structure will be resilient to water limitations in an agroforest but not in a monoculture.

## 2 Materials and Methods

### 2.1 Site description

Our experiment was conducted from June to September 2014 at the University of Guelph Agroforestry Research Station, which is a long‐term agroforestry research site established in 1987 on 30 ha of agricultural land in Guelph, Ontario, Canada (43°32′N, 80°12′W). The agroforestry system maintains rows of 17 different tree species, all planted in 1987, whereby tree rows are spaced 12.5–15 m apart and individual trees within a row are spaced at 3–6 m (Borden, Isaac, Thevathasan, Gordon, & Thomas, 2014). Since 1991, soybean, corn (*Zea maize* L.), barley (*Hordeum vulgare* L.), and wheat (*Triticum aestivum* L.) (Peichl, Thevathasan, Gordon, Huss, & Abohassan, 2006) have been planted in the alleys between the tree rows as sole crops that are rotated annually. The research site also maintains a paired monoculture system site, which is located directly adjacent to the agroforestry system, situated approximately 300 m southwest from the eastern boundary of the agroforestry system. Beyond the presence or absence of trees, crops in both the conventional monoculture and agroforestry system are managed under the same management regime, which entails no‐till cultivation, zero fertilizer inputs, rain‐fed irrigation, and the same crop rotation.

Our study was therefore based in two different systems, which were defined by the management employed: (1) agroforest and (2) monoculture. For our experiment, soybean (variety: Pioneer P90Y90) was planted in both the agroforest and monoculture at a seeding rate of approximately 450,000 seeds ha^−1^ (7.5 in. row spacing). In both systems, glyphosate‐based pesticides were applied, but these pesticides were explicitly excluded from the area within our experimental plots (defined in detail below).

A weather station located approximately 28 km from the experimental site provided long‐term climate information. During the experiment, the average weekly maximum and minimum temperature was 27.3 and 6.0°C, respectively, while average monthly rainfall was 78.25 mm. Compared to historical averages from 1980 to 2010, the site experienced more than average rainfall in July 2014 (157 vs. 89 mm average) and lower than average rainfall in both June 2014 (66 vs. 83 mm average) and August 2014 (65 vs. 97 mm average).

Over the course of the experiment, air temperature, relative humidity, and photosynthetically active radiation (PAR) were monitored with data loggers (Onset HOBO‐USA), taking measurements at 30‐minute intervals. On average across four data loggers, air temperature in the monoculture and agroforest was recorded as 17.5 ± 0.4 and 17.7 ± 0.4°C, respectively, relative humidity as 86.8 ± 0.7% and 86.5 ± 4.0%, respectively, and PAR as 529 ± 46 and 222 ± 14 μmol m^−2^ s^−1^, respectively. Gravimetric soil moisture under full rainfall at the beginning of the experiment in the monoculture and agroforest was 19.0 ± 0.4% and 16.2 ± 0.3%, respectively.

Soils at the site are classified as Gray Brown Luvisols with a sandy‐loam soil texture (65% sand, 25% silt, and 10% clay; Order: Alfisols, Group: Typic Hapludalf) (Oelbermann & Voroney, 2007). A detailed analysis of the soil physical and chemical properties at our study site can been found in Lacombe, Bradley, Hamel, and Beaulieu (2009), but briefly for the monoculture and agroforest, respectively, total base cations at the site are 8.76 and 9.57 mg Ca^+^ g^−1^; 2.84 and 3.05 mg K^+^ g^−1^; 5.17 and 8.49 mg Mg^+^ g^−1^; and 1.69 and 1.20 mg Na^+^ g^−1^ (Lacombe et al., 2009). Soil phosphate at the site was measured as 30.71 ± 2.55 and 32.65 ± 1.58 mg PO^3−^_4_ kg^−1^ for the monoculture and agroforest, respectively. Soil nitrate at the site was measured as 15.7 ± 2.35 and 15.76 ± 1.86 mg NO^−^_3_ kg^−1^ for the monoculture and agroforest, respectively, and soil ammonium as 5.30 ± 0.97 and 9.28 ± 6.10 mg NH^+^_4_ kg^−1^ for the monoculture and agroforest, respectively.

Based on previous work at the same experimental site, the percentage of N derived from atmosphere (%Ndfa), measured from soybean leaves, was significantly higher in the agroforest as compared to the monoculture (Nasielski et al., 2015). Specifically, at the “V5” (vegetative) stage of annual soybean growth, %Ndfa was reported as 2.7 ± 0.9 and 64.3 ± 4.1 for the monoculture and agroforest, respectively. At the “R3” (beginning pod) stage of soybean growth, %Ndfa was reported as 49.7 ± 8.0 and 77.0 ± 4.0 for the monoculture and agroforest, respectively. Finally, at the “R6” (seed fill) stage, %Ndfa in soybean was reported as 65.0 ± 8.0 and 94.3 ± 4.7 for the monoculture and agroforest, respectively (Nasielski et al., 2015).

### 2.2 Experimental design

To test the effects of water limitation and management on general fungal and AMF community structure in the roots of soybeans, we employed a split‐plot design, whereby agroforest and monoculture were considered the whole plot effect. For each of these management systems, we delineated six replicated blocks. Of the tree species present in the agroforest, silver maple (*Acer saccharinum* Marsh.) trees were selected as the alley row tree species to adjacently place all agroforestry plots.

**Figure 2:**
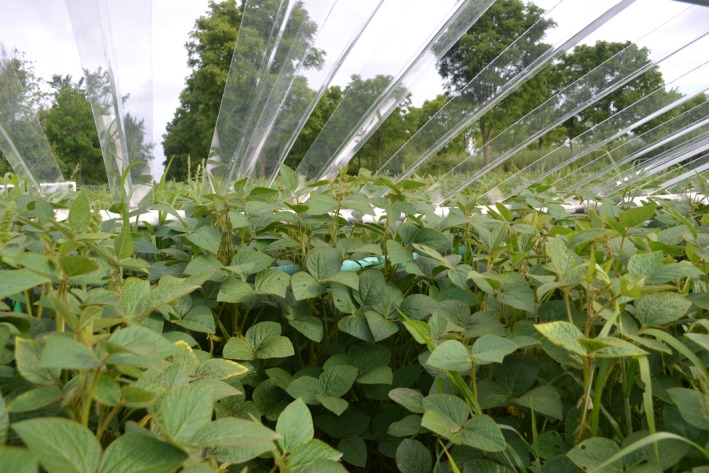
*In situ* rainfall reduction shelter at the University of Guelph Agroforestry Research Station (Guelph, Ontario, Canada)

Within each block, we assigned the rainfall treatment, which entailed (1) a full rainfall and (2) reduced rainfall treatment. Rainfall reduction was simulated in situ using rainfall reduction shelters, which were designed based on those previously employed in studies of crop drought response and were effective in reducing incoming precipitation by 50%–80% (Yahdjian & Sala, 2002; Gherardi & Sala, 2013). Specifically, the rainfall shelters measured 2.5 × 1.1 m (2.75 m^2^) constructed of v‐shaped, transparent, acrylic troughs that diverted rainfall off the area beneath the shelter (Figure 2). In the agroforestry plots, shelters were placed in each block over soybean growing 2 m away from the tree row, directly adjacent to a tree trunk, but under the tree canopy drip line.

Shelters were deployed 1 week after soybean emergence and remained for 6 weeks. Among the monoculture plots, rainfall reduction shelters resulted in a 12.8% difference in soil moisture, 0.2% difference in relative humidity, 0.6% difference in temperature, and an 8.4% difference in PAR as compared to the full rainfall plots. Among the agroforestry plots, rainfall reduction shelters resulted in a 16.2% difference in soil moisture, 2.8% difference in relative humidity, 2.7% difference in temperature, and a 8.6% difference in PAR as compared to the full rainfall plots. This 12.8% and 16.2% decline in soil moisture under rainfall shelters aligns closely with projected 10%–20% reductions in precipitation under future climate scenarios in Southern Ontario (Environmental Commissioner of Ontario 2008). Seven weeks after shelters were removed, gravimetric soil moisture returned to 89.3% of the full rainfall plots in the monoculture to 98.5% of full rainfall plots in the agroforest.

### 2.3 Plant sampling

In order to understand the response of fungal community structure to water limitation, sampling was conducted during and after rainfall shelters were removed (Figure 1). Root samples were taken on two different sampling dates from both the monoculture and agroforest: (1) while rainfall shelters were in place for 6 weeks during the “R2” or flowering stage of soybean development (as it has been demonstrated that mycorrhizal infection is highest during this stage; Abdel‐Fattah, 1997) termed “stress,” and (2) after rainfall shelters were removed, at the “R7” or beginning maturity stage of soybean development (7 weeks after rainfall shelter removal) termed “poststress.” The “no stress” samples were taken at the same time as the “stress” samples but under full rainfall in order to minimize the effects of shifts in fungal communities during soybean establishment.

Plants were sampled from within a 25‐cm^2^ quadrat, both under the rainfall shelter and adjacent full rainfall plots. Individual plants were carefully excavated from the soil by hand, with care taken to ensure the entire rooting structure was extracted completely intact. Immediately following collection, all root samples were stored at 4°C and were transported to the University of Toronto, Scarborough, for analysis, where they were stored at −20°C until DNA processing.

### 2.4 DNA extraction

Prior to DNA extractions, roots were washed thoroughly in distilled water and root sections were removed using an ethanol‐sterilized scalpel. Extractions were then performed on 50 mg of root tissue, using PowerPlant DNA Isolation Kit and following published protocols (MO BIO Laboratories Inc., Carlsbad, CA, USA). Following the extraction process, total DNA concentration in all samples was estimated spectrophotometrically using a NanoDrop ND‐1000 V3.7.0 spectrophotometer (Thermo Fisher Scientific Inc., Wilmington, DE, USA) and visualized by gel electrophoresis on a 0.8% (wt/vol) agarose gel containing RedSafe Nucleic Acid Staining Solution (FroggaBio Inc., North York, ON, CA) in 0.5% Tris/Borate/EDTA buffer and quantified using a DNA ladder (GeneRuler 1 kb DNA Ladder Plus, Fermentas, Burlington, Ontario, CA). All gels were run at 100 V for 30 min until distinct bands were resolved. The average yield of DNA was 19.2 ngμl^‐1^ for soybean root samples.

### 2.5 Amplification and digestion of general fungal fragments

Primers designed to specifically amplify fungal sequences for the intertranscribed spacer (ITS) region of the small ribosomal operon (ITS1F 5′‐CTT GGT CAT TTA GAG GAA GTA A‐3′ forward and ITS4 5′‐TCC TCC GCT TAT TGA TAT GC‐3′ reverse) were used to detect general fungal colonizers (Manter & Vivanco, 2007). General fungal sequences were specifically used as a comparison to AMF sequences. All primers were labeled with fluorescent dyes, phosphoramidite 6‐FAM and HEX (forward and reverse labeled at the 5′ end, respectively; Invitrogen, Canada). Twenty μl amplification reactions consisted of 10 μl of HotStar Taq Plus Master Mix (Qiagen, CA), 1 μl of each primer at 10 μmolL^‐1^, 7 μl of RNA‐free water, and 1 μl of DNA template. Reactions were incubated in a PTC‐100 thermal cycler (MJ Research Inc., Waltham, Massachusetts, USA) under the following conditions: DNA polymerase initialization at 95°C for 5 min, followed by 34 cycles at 94°C for 50 s, 51°C for 1 min, ending with a final extension at 72°C for 10 min. Twenty μl RNA‐free water was run as a negative control. Amplicons were digested using the restriction enzymes *Eco*RII and *Fsp*BI for 2 hr at 37°C (Fermentas Canada Inc., Burlington, Ontario, CA, USA). Digests contained 15 μl of PCR product, 2U of each *Eco*RII and *Fsp*BI, 2 μl Tango 10 ×  buffer, and 2.6 μl RNA‐free water. ITS amplicons of approximately 65–2255 bp in length were successfully obtained from root DNA.

### 2.6 Amplification and digestion of AMF fragments

Primers designed by Lee, Lee, and Young (2008) to amplify the small ribosomal subunit (SSU) (AML1 5′‐ATC AAC TTT CGA TGG TAG GAT AGA‐3′ forward and AML2 5′‐GAA CCC AAA CAC TTT GGT TTC C‐3′ reverse) were used for amplifying AMF exclusively (Phylum: Glomeromycota). All primers were labeled with fluorescent dyes phosphoramidite, 6‐FAM and HEX (forward and reverse labeled at the 5′ end, respectively; Invitrogen, CA). Twenty μl amplification reactions consisted of 10 μl of HotStar Taq Plus Master Mix (Qiagen, CA), 1 μl of each primer at 10 μmolL^‐1^, 7 μl of RNA‐free water, and 1 μl of DNA template. Reactions were incubated in a PTC‐100 thermal cycler (MJ Research Inc., Waltham, Massachusetts, USA) under the following conditions: DNA polymerase initialization at 95°C for 50 s, followed by 34 cycles at 95°C for 50 s, 55.5°C for 50 s, 72°C for 60 s, ending with a final extension at 72°C for 10 min. 20 μl RNA‐free water was run as a negative control. Amplicons were digested using the restriction enzymes *Alu*I and *Hinf*I for 2 hr at 37°C (Fermentas Canada Inc., Burlington, Ontario, CA). Digests contained 15 μl of PCR product, 2U of each *Alu*I and *Hinf*I, 2 μl Tango10X buffer, and 2.6 μl RNA‐free water. AML amplicons of approximately 60–812 bp in length were successfully obtained from root DNA.

### 2.7 Terminal restriction fragment analysis of TF and AML fragments

Each sample was then analyzed at the University of Guelph, using a 3730 DNA sequencer (Applied Biosystems Inc., Fremont, CA, USA) for sizes and intensities (i.e., peak height) of the 5′‐terminal fragment. DNA sequence polymorphisms are used to classify diversity in terms of phylotypes. In other terms, the T‐RFLP approach does not measure fungal diversity *per se*, but rather the number of individual fluorescent peaks in a sample, which in turn correspond to different fungal phylotypes. Signals with a peak height below 110 relative fluorescent units were regarded as background noise and excluded from analysis (Lueders & Friedrich, 2003). Fragment sizes ranged from 60 to 600 base pairs (bp). In sum, for analysis the total number of terminal restriction fragments (TRF) was treated as an estimate of the fungal community diversity.

### 2.8 Statistical analysis

All analyses were performed using R v. 3.1.2 (The R Foundation for Statistical Computing, Vienna, Austria). We first used analysis of variance (ANOVA) to test for differences in soil P, soil moisture, relative humidity, temperature, and PAR across treatments.

Peak height data for each individual TRF was first normalized prior to analysis (Fredriksson, Hermansson, & Wilén, 2014) and exported for analysis using R. We used nonmetric multidimensional scaling (NMDS) ordination analysis in the “*vegan”* R package (Oksanen, 2015), to describe differences in fungal community structure. NMDS is commonly used to describe variation in T‐RFLP data, as it is a nonparametric procedure that preserves ranked differences among peaks (Rees, Baldwin, Watson, Perryman, & Nielsen, 2004). For all NMDS analysis, we first used a Wisconsin double standardization to standardize all data (Oksanen, 2015). We then constructed a global community matrix among all of our samples, based on Bray–Curtis distances (Bray & Curtis, 1957), and used this matrix as the basis to calculate pairwise compositional dissimilarities between any two samples as:

$$BC_{jk}=\frac{\sum i\;\left|x_{ij}-x_{ik}\right|}{\sum i\;\left(x_{ij}+x_{ik}\right)}$$

where *BC*_*jk*_ represents the Bray–Curtis dissimilarity between the *j*th and *k*th sample, *x*_*ij*_ represents the abundance of taxon *i* in sample *j*, and *x*_*ik*_ represents the abundance of taxon *i* in sample *k*. The NMDS analysis returns a “stress” value for the overall model fit, which ranges between 0 and 1 and is interpreted as a measure of fit of the multivariate data, with smaller values indicating a better overall fit (Oksanen, 2015).

Differences in community structure among experimental factors were then evaluated using an Adonis test, which is analogous to a multivariate analysis of variance, and partitions the variation in community dissimilarities across (in our case) management treatments, rainfall treatments, and time (sample dates), as well as all two‐ and three‐way interactions among these variables. In our analysis, this test returns the variance (*r* ^2^) in both AMF and general fungal community structure (analyzed separately) explained by different treatments and interactions, and employs permutations tests to generate a randomized null distribution of *F*‐statistics; type 1 error rates for each experimental level are then generated by comparing the observed *F*‐value to the distribution of randomized *F*‐value (Foster et al., 2011). In order to visualize differences in community structure, we also calculated and present 95% confidence limits surrounding NMDS scores for a given generalist or AMF community within different management, rainfall, or time treatments.

Since we were explicitly interested in understanding how management moderates the resistance and resilience of fungal community structure to water limitation, and as our first analyses indicated significant interactions between management and both rainfall and time of sampling (see Section 3), we conducted a second Adonis test on subsets of our data. Specifically, we used the same test to evaluate the effects of rainfall and time on both generalist and AMF community structure, in both agroforestry and monoculture separately. These models did not include interaction effects.

## 3 Results

### 3.1 Effect of rainfall reduction on general fungal and AMF community structure

**Figure 3:**
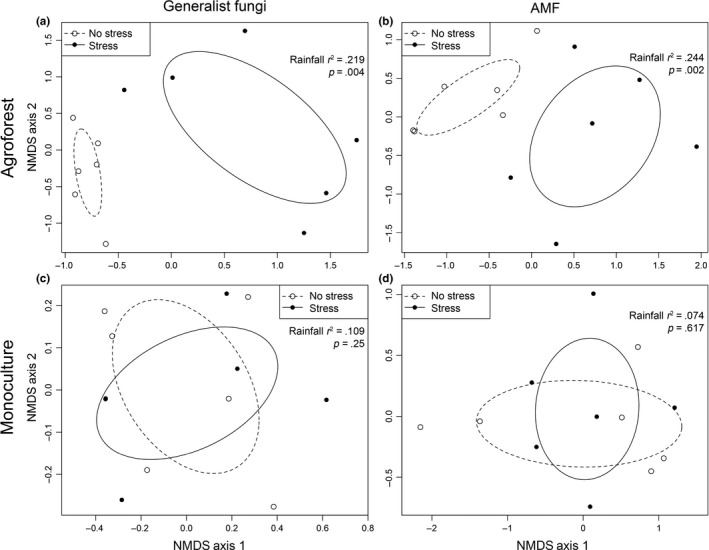
Nonmetric multidimensional scaling (NMDS) ordinations for fungal communities in an agroforest (panels a and b) and monoculture (panels c and d). Plots are derived from normalized TRFs obtained from soybean root samples, organized by rainfall treatment (open circles represent full rainfall sites (no stress) and filled circles represent rainfall reduction sites (stress)). Data are shown for both generalist fungal communities (panels a and c) and AMF communities (panels b and d). For each panel, *r* ^2^ and *p‐*values as well as 95% confidence ellipses surrounding each grouping are provided

Rainfall reduction treatments did not result in significant differences between general fungal community structure (*p *= .103) or AMF community structure (*p *=* *.092; Table 1) when samples from both the monoculture and the agroforest were combined. Specifically, rainfall treatments only explained 2.5% and 3.1% of the observed variation in generalist fungi and AMF, respectively (Table 1). However, the effects of rainfall reduction on fungal community structure were detected in the agroforest but not in the monoculture. Across rainfall treatments in the agroforest, both general fungal (*p *=* *.042) and AMF (*p *=* *.004) communities differed significantly (Table 2, Figure 3a,b). In the monoculture, however, neither general fungal (*p *=* *.693) nor AMF (*p *=* *.588) phylotypes differed significantly as a function of rainfall reduction (Table 2). Visually, there was a strong overlap in 95% confidence intervals for phylotypes sampled under full rainfall and rainfall reduction treatments (Figure 3c,d).

**Table 1:** Variation in fungal community composition as a function of management, rainfall treatments, and time, in a Southern Ontario agroecosystem

Variable	General fungal	AMF
Management	**0.048 (0.001)**	**0.053 (0.005)**
Time	**0.079 (0.001)**	**0.048 (0.008)**
Rainfall treatment	0.025 (0.103)	0.031 (0.092)
Management × time	**0.035 (0.007)**	0.017 (0.510)
Management × rainfall treatment	0.021 (0.267)	**0.037 (0.030)**
Time × rainfall treatment	0.033 (0.013)	0.015 (0.678)
Management × time × rainfall treatment	**0.043 (0.001)**	0.033 (0.060)
NMDS stress	0.25	0.17

Results are based on a permutational multivariate analysis of variance (Adonis test) for both general fungal phylotype, and AMF phylotype communities. Presented are *r* ^2^ values, interpreted as the explained variation for each independent variable or interaction effect (denoted by “*”). Values in brackets represent *p*‐values based on permutation tests, and significant values (where *p *<* *.05) are highlighted in bold. Also presented for each fungal group is a descriptive stress value associated with the NMDS procedure, where lower values generally indicated a better NMDS model fit (see section 2).

**Table 2:** Variation in fungal community composition as a function of rainfall treatments and time, in agroforestry and monoculture systems

Management treatment	Fungal group	Rainfall	Time	Stress
Monoculture	General fungal	0.033 (0.693)	**0.118 (<0.001)**	0.193
	AMF	0.036 (0.588)	**0.076 (0.044)**	0.135
Agroforestry	General fungal	**0.063 (0.042)**	**0.120 (0.001)**	0.226
	AMF	**0.115 (0.004)**	0.06 (0.112)	0.180

Results are based on permutational multivariate analysis of variance (Adonis test) for general fungal phylotypes and for AMF phylotypes. Values represent *r* ^2^ values, interpreted here as the explained variation for each independent variable or interaction effect, and values in brackets represent *p*‐values based on permutation tests. Significant values (where *p *<* *.05) are highlighted in bold. Also presented is a descriptive stress value associated with the NMDS procedure performed for each dataset, where lower values generally indicated a better NMDS model fit (see section 2).

### 3.2 General fungal and AMF community structure between management systems

**Figure 4:**
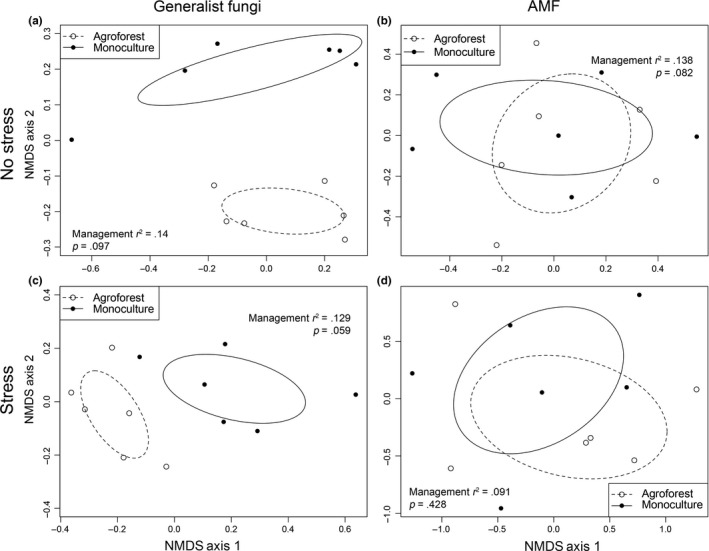
Nonmetric multidimensional scaling (NMDS) ordinations for fungal communities under full rainfall (no stress; panels a and b) and under rainfall reduction (stress; panels c and d). Plots are derived from normalized TRFs obtained from soybean root samples, organized by management system (open circles represent agroforests and filled circles represent monocultures). Data are shown for both generalist fungal communities (panels a and c) and AMF communities (panels b and d). For each panel, *r* ^2^ and *p‐*values as well as 95% confidence ellipses surrounding each grouping are provided

The management system did not result in significant differences between AMF community structure (*p *=* *.060) when samples were controlled for time of sampling and rainfall treatment (Table 1). However, general fungal community structure in soybean roots did significantly vary (*p *=* *.001) between management systems when controlling for time of sampling and rainfall treatment (Table 1). Visually, there is no overlap in the phylotypes sampled under agroforest and monoculture for both no stress and stress treatments (Figure 4a,c).

### 3.3 General fungal and AMF community structure postrainfall reduction

**Figure 5:**
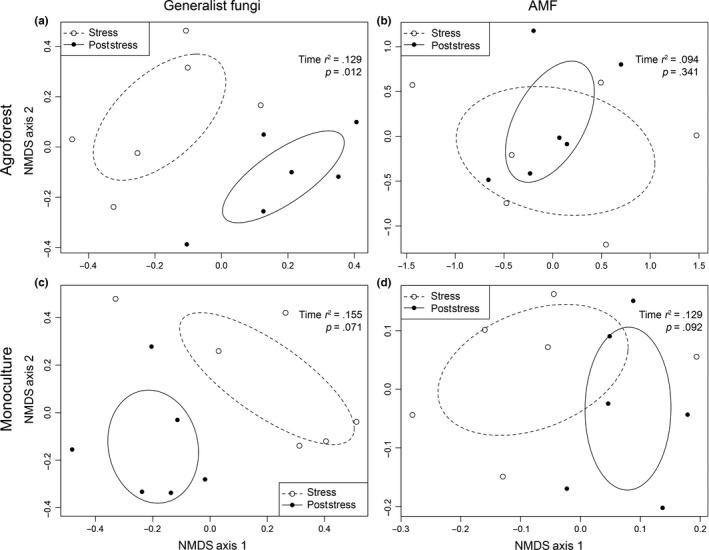
Nonmetric multidimensional scaling (NMDS) ordinations for fungal communities in an agroforest (panels a and b) and monoculture (panels c and d). Plots are derived from normalized TRFs obtained from soybean root samples, organized by time (open circles represent rainfall reduction sites (stress) and filled circles represent rainfall reduction removal (poststress)). Data are shown for both generalist fungal communities (panels a and c) and AMF communities (panels b and d). For each panel, *r* ^2^ and *p‐*values as well as 95% confidence ellipses surrounding each grouping are provided

Across our entire dataset, soybean root samples collected during rainfall reduction and seven weeks after rainfall reduction removal, exhibited significantly different general fungal (*p *=* *.001) and AMF (*p *=* *.008) communities (Table 1). Whether or not communities were sampled during or after the removal of rainfall reduction shelters explained 7.9% and 4.8% of the variation in generalist and AMF community structures, respectively (Table 1). Specifically, in both the agroforest and monoculture, general fungal communities were significantly different (*p *=* *.001, *p *<* *.001, respectively) under rainfall reduction and rainfall reduction removal (Table 2, Figure 5a,c); rainfall reduction removal explained approximately 12.0% and 11.8% of the variability in phylotypes, respectively (Table 2). However, AMF phylotypes in the agroforest and monoculture did not show the same trend: in both systems, there was no significant (*p *=* *.112) to little significant (*p *=* *.044) difference, respectively, in AMF communities under rainfall reduction and after rainfall reduction removal (Table 2, Figure 5b,d). In this case, only 6.0% and 7.6% of the variability in AMF communities was explained, respectively.

## 4 Discussion

In our study, water limitation affected both general fungal and AMF communities in soybean roots cultivated in an agroforestry system (Figure 3a,b; Table 2). However, this was not observed in soybean roots for plants growing in monoculture (Figure 3c,d). Our data indicate that generalist fungi and AMF in soybean roots in monoculture systems are better able to resist immediate changes in soil moisture, as compared to fungal communities in agroforestry systems. Although we expected that AMF community structure would change under both management systems, our results did not confirm our original hypothesis that the impacts of water limitation would be observed in both systems. However, this response may be strain dependent. For example, Davies et al. (2002) demonstrated that water stress reduced colonization by one *Glomus* sp. strain while another *Glomus* sp. strain showed enhanced arbuscule formation and hyphae development under water stress. More generally, in an earlier review, Augé (2001) found that drought affected fungal root colonization in only about half of published studies, but increasing root colonization was observed more often than decreasing root colonization.

Under short‐term water limitation, however, general fungal and AMF community structure may not be affected as a result of adaptations of certain fungal species to water stress conditions, which may have been present only in the monoculture system. For example, *Glomus intraradices* and *Glomus deserticola* are generally regarded as less inhibited by water stress, as compared to *Glomus etunicatum* (Augé, 2001), and thus, some AMF may resist water stress better than others (Compant et al., 2010). In a study by Börstler, Thiéry, Sýkorová, Berner, and Redecker (2010), haplotype richness of *Glomus intraradices* was found to be higher in tilled agricultural sites when compared to species‐rich seminatural grasslands. Picone (2000) also suggested that *Glomus* sp. are flexible in terms of their response to environmental variability and are able to adjust sporulation or colonization in order to tolerate unfavorable conditions.

Our results indicate that both general fungi and AMF sampled from soybean roots in the agroforestry system are more sensitive to immediate short‐term water limitation as compared to the monoculture system. It has been shown that agroforestry or “more naturalized” systems promote a higher diversity of AMF as compared to conventional monocultures (Cardoso, Boddington, Janssen, Oenema, & Kuyper, 2003; Muleta, Assefa, Nemomissa, & Granhall, 2008; Chifflot et al., 2009; Lacombe et al., 2009), although this diversity may be spatially related to the species of tree in an agroforestry system (Bainard et al., 2011). It is possible that generalist, non‐Glomeromycotan fungi are associating with soybean roots as well, possibly out‐competing Glomeromycotan fungi. Such patterns may owe to the fact that AMF are nonhost specific (Bever, Morton, Antonovics, & Schultz, 1996; Rillig, Wright, & Eviner, 2002; Kumar, Shukla, Hashmi, & Tewari, 2007; Smith & Read, 2008; Shukla, Kumar, Jha, Dhyani, & Vyas, 2012). In turn, the establishment of new taxa within the fungal community may be difficult, especially when new species are competing with well‐adapted local communities (Verbruggen, van der Heijden, Rillig, & Kiers, 2013). Under these scenarios, competitive exclusion of functionally similar AMF species may prevent the co‐occurrence of closely related species that are well adapted to water limitation, such as those in an agroforestry system. Further sequencing would be needed to determine exact members of the community.

It is also possible that the water reduction was more intense in the agroforestry system as water competition between trees and crops may occur. Although there is some evidence that water competition is limited and seasonally variable in these agroforestry systems (Link, Thevathasan, Gordon, & Isaac, 2015), further reduction through our treatments may have caused a less resistant fungal community. Therefore, the aboveground microclimatic buffering of agroforestry systems may be offset by soil water competition and thus may result in highly sensitive fungal communities.

In agroforestry and monoculture systems, the community structure of generalist fungi sampled during water reduction and after the removal of water reductions differed significantly (Table 2). Therefore, in the agroforestry systems, soybean root general fungal communities were significantly different during water limitation and after water limitation was removed. Given our conceptualization (Figure 1), these changes in community structure indicate that generalist fungi show a capacity for resilience to water limitation in the agroforestry system. Alternatively, these changes in poststress fungal communities may indicate a continued change away from the initial community state. However, we are restricted in our identification of species within these communities given our T‐RFLP data, and thus, this change may represent only a partial “recovery” of community composition that was present before stress was applied. It is also possible that community composition may have changed concomitantly with the progression of the season; however, there are mixed results of seasonality effects on AMF community composition (Rosendahl & Stuckenbrock, 2004; Santos‐González, Finlay, & Tehler, 2007). The lack of consensus across studies examining seasonality effects on community composition is concerning; however, sampling in the present study was consistent across treatments, suggesting that this detected change in general fungal community structure is a response in the population to water limitation removal.

In both management systems, AMF community structure in soybean roots was not significantly different during rainfall reduction and after rainfall reduction was removed (Table 2). This is where the two systems diverge. In monoculture, no difference was found for AMF community structure during water stress and after water stress removal. Thus, the data are insufficient to clearly invoke resiliency as a generalizable ecological feature of these systems. However in the agroforestry system, the AMF community structure was affected by rainfall reduction and then showed no change after rainfall reduction removal. This would suggest that root AMF is not resilient (Figure 1), such that we detect no difference in the community structure, and therefore no response, to the removal of water stress.

Given that trees in an agricultural system provide modifications to air temperature, water vapor content, relative humidity, PAR, and wind velocity (Jose, Gillespie, & Pallardy, 2004; Karki & Goodman, 2013), and stimulate higher biodiversity in both above‐ and belowground flora and fauna (Bainard et al., 2012; De Beenhouwer, Aerts, & Honnay, 2013), we expected the agroforestry systems to contribute to resistance and resilience of AMF to water limitation. While it has been shown that trees and crops can harbor the same species of AMF (Ingelby, Wilson, Munro, & Cavers, 2007), further research is needed to assess to the role of trees in maintaining AMF and other beneficial organisms (Lacombe et al., 2009; Pierantozzi et al., 2014). Finally, the spatial‐temporal dynamics of AMF community structure still remain largely unknown (Dumbrell et al., 2011).

## 5 Conclusions

Our findings represent some of the first field data that characterizes the resistance and resilience of fungal communities to water limitation in a temperate agroecosystem. Our work also contributes to the increasing effort to investigate the response of soil microorganisms to environmental change. Given that the structure and dynamics of fungal community are limited here to the interpretation of T‐RFLP data, it appears that water limitation has an effect on both generalist fungi and AMF in agroforestry systems, but not in monoculture systems. Overall, general fungal communities are more resilient to water limitation than AMF, specifically in the agroforestry system. Future research examining fungal colonizers under different environmental stressors, and which abiotic stressors have the largest influence on fungal diversity, is needed. More broadly, a better understanding of how the influence of abiotic stressors on fungal communities varies across cropping regions or climatic zones would have immediate widespread implications for understanding agroecosystem responses to global change.

## Conflict of interest

None declared.
